# Tetramethylpyrazine Alleviates Behavioral and Psychological Symptoms of Dementia Through Facilitating Hippocampal Synaptic Plasticity in Rats With Chronic Cerebral Hypoperfusion

**DOI:** 10.3389/fnins.2021.646537

**Published:** 2021-05-06

**Authors:** Zihu Tan, Jing Qiu, Yuting Zhang, Qiong Yang, Xixi Yin, Jia Li, Guangya Liu, Hengfei Li, Guang Yang

**Affiliations:** ^1^Department of Geriatrics, Hubei Provincial Hospital of Traditional Chinese Medicine, Wuhan, China; ^2^Hubei Provincial Academy of Traditional Chinese Medicine, Wuhan, China; ^3^Clinical College of Traditional Chinese Medicine, Hubei University of Chinese Medicine, Wuhan, China; ^4^The First Clinical College, Hubei University of Chinese Medicine, Wuhan, China; ^5^College of Acupuncture and Orthopedics, Hubei University of Chinese Medicine/Hubei Provincial Collaborative Innovation Center of Preventive Treatment by Acupuncture and Moxibustion, Wuhan, China; ^6^Department of Infectious Diseases, Hubei Provincial Hospital of Traditional Chinese Medicine, Wuhan, China

**Keywords:** behavioral and psychological symptoms of dementia, tetramethylpyrazine, chronic restraint stress, neurodegenerative disorders, synaptic remodeling, TrkB/ERK/CREB signaling pathway

## Abstract

Behavioral and psychological symptoms of dementia (BPSD) ubiquitously disturb all patients with dementia at some point in the disease course. Although a plethora of non-pharmacological and pharmacological methods targeting the relief BPSD have been developed, the therapeutic effect is still far from ideal. Here, a rat BPSD model combining the physiological changes with mental insults was successfully established. Meanwhile, our results indicated that TMP attenuated anxious behavior using an elevated plus maze (EPM) test, ameliorated recognitive ability and sociability through a novel object recognition test (NORT) and social interaction test (SIT), and improved learning and memory impairments *via* a Barnes maze in rats with bilateral common carotid arteries occlusion (BCCAO) plus chronic restraint stress (CRS). Given that hippocampus chronic cerebral hypoperfusion (CCH) always causes damage to the hippocampus, and the majority of cognitive impairments, behaviors, and stress responses are associated with pathology in the hippocampus including anxiety and depression, we paid attention to investigate the role of the hippocampus in BPSD. Our results indicated that Tetramethylpyrazine (TMP) attenuated anxiety and ameliorated recognitive ability, sociability, learning, and memory impairments due to alleviating dendritic and spine deficits, and upregulating the expression of synapse-related proteins (including PSD95, SYN, GAP43, SYP) in the hippocampus. We also found that the underlying mechanism was that TMP could activate the TrkB/ERK/CREB signaling pathway to promote synaptic remodeling *in vivo* and *in vitro*. Mechanically, the present study enlarges the therapeutic scope of TMP in neurodegenerative disorders and provides basic knowledge and feasible candidates for treating BPSD, particularly for vascular dementia.

## Introduction

Dementia is a progressive neurodegenerative disorder causing both functional and cognitive deficits (Cascade et al., 2008; Matthews et al., 2013). Notwithstanding the prevalence and incidence of dementia exhibiting a decreased tendency during recent decades (Wu et al., 2017), the behavioral and psychological symptoms of dementia (BPSD) are ubiquitous in patients with dementia, affecting 80–100% of individuals at some point during the disease course (Manso-Calderón et al., 2020). The symptoms of BPSD include, but are not limited to, physical aggression, screaming, restlessness, anxiety, depression, apathy, agitation, aberrant motor behavior, sexual disinhibition, hallucinations, delusions, and sleep and appetite disturbances (Pan et al., 2013; Kales et al., 2015; Bessey and Walaszek, 2019). Furthermore, these distractions always result in accelerated cognitive and functional deterioration, declined quality of life, and elevated mortality in sufferers with dementia, which impose higher burdens for caregivers, high risk of institutionalization, and higher costs of care (Finkel, 2001). Thereafter, exploring feasible approaches for treating BPSD not only benefits patients with dementia, but also reduces socioeconomic burden, to some extent.

A plethora of non-pharmacological and pharmacological methods targeting the relief of BPSD have been developed (Kales et al., 2015). Non-pharmacological interventions, such as cognitive stimulation training, exercise, music therapy, light therapy, and aromatherapy exert some benefits and have been recommended as the first line therapeutic strategy for BPSD by a series of guidelines, experts, and medical organizations (American Geriatrics Society and American Association for Geriatric Psychiatry, 2003; National Collaborating Centre for Mental Health, and National Institute for Health and Clinical Excellence: Guidance, 2007; Kales et al., 2014). Although these strategies provide a beautiful blueprint for patients, they are restricted from being used in practice by the lack of experienced care givers, insufficient training, poor family understanding of mental health problems, and/or underdiagnosis of psychiatric problems in primary care settings (Molinari et al., 2010; Kales et al., 2015). Furthermore, studies have represented some adverse events associated with non-pharmacological strategies, such as enhanced agitation with cognitive or emotional oriented interventions, and increased agitation and physical aggression for sensory strategies such as music therapy, massage, and touch therapies, and aromatherapy (Cooke et al., 2010; O’Neil et al., 2011; Kales et al., 2015). Herein, pharmacological approaches, including typical and atypical antipsychotics, have been widely used for the treatment of BPSD. However, these antipsychotic drugs occasionally result in adverse events including extrapyramidal symptoms (parkinsonism, dystonia, and tardive dyskinesia) and other side effects, such as postural hypotension, anticholinergic effects, hyperprolactinemia, weight changes, sexual dysfunction, glucose dysregulation, seizures, and falls (Reilly et al., 2000; Schneider et al., 2006; Ballard and Corbett, 2010; Kales et al., 2015). Therefore, the United States Food and Drug Administration (FDA) has twice warned that both conventional and atypical antipsychotics are associated with an increased risk of mortality in elderly patients with dementia-related psychosis in 2005 and 2008 (Cascade et al., 2008; Howland, 2008; Jeste et al., 2008; Rose and Kass, 2019). Derivatives extracted from Chinese herbal medicines might provide a suitable and safe candidate for improving BPSD, as well as avoiding side effects for a long-time treatment using antipsychotics.

Tetramethylpyrazine (TMP) is one of the active ingredients extracted and purified from a traditional Chinese herb medicine Ligusticum wallichii Franchat (Chuanxiong), which has been universally administrated for patients with post-stroke symptoms throughout the long history of China (Zhao et al., 2018). Recent studies have indicated that TMP and its derivatives could serve as antioxidants to rescue neuronal loss and improve behavioral performance in animal models of Alzheimer’s disease (AD) (Shi et al., 2012; Pu et al., 2015). Meanwhile, investigations reveal that TMP analogs CXC195 and DT-010 protect dopaminergic neurons against apoptosis through the activation of PI3K/Akt/GSK3β signaling pathway in neurodegenerative disease models, including Parkinson’s Disease (PD) and AD (Chen et al., 2017; Hu et al., 2018; Zhou et al., 2019). Moreover, TMP could reduce microglial activation and accumulation of pro-inflammatory mediators in response to inflammatory stimuli or Amyloid β peptide (Aβ) in neurodegenerative diseases (Kim et al., 2014; Pu et al., 2015). In addition, research has demonstrated that TMP analog tetramethylpyrazine nitrone (TBN) reduces cortical and hippocampal deficits and white matter lesions, potentiates axonal outgrowth, and attenuates oxidative damage through PI3K/Akt/GSK3b signaling pathway, resulting in improved cognitive impairment in a rat chronic hypoperfusion model, which is widely utilized to determine the role of cerebral hypoperfusion in neurodegenerative processes including vascular dementia and AD (Zhang et al., 2017). Most recently, research has indicated that synaptic plasticity plays an evident role in depression-like behavior and could serve as a therapeutic target for BPSD (Castrén, 2005; Duman et al., 2016). With respect to TMP and its derivatives holding multifunctional neuroprotective effects in neurodegenerative diseases, the therapeutic effect of TMP on BPSD still remains elusive. Hence, it is worthy to explore the role of TMP in treating BPSD and decipher its underlying mechanism.

In the present study, we speculated that TMP could benefit BPSD through promoting hippocampal synaptic plasticity in rats. The aim of the present study is to enlarge the therapeutic scope of TMP in neurodegenerative disorders, and to provide basic knowledge and feasible candidates for treating BPSD.

## Materials and Methods

### Animals

The present research was approved by Hubei Provincial Hospital of Traditional Chinese Medicine and all procedures were carried out according to the Chinese Animal Welfare Legislation for protection of animals used for scientific purposes. Every effort was made to reduce animal suffering and to minimize the number of animals used in the present work. A total of 140 male adult Sprague Dawley (SD) rats were obtained from Experimental Animal Research Center of Hubei Province. All rats were housed on a constant photoperiod (12-h light/dark cycle), moisture (55–60%), and temperature (22 ± 3°C) condition. All rats were provided food and water *ad libitum* before and after surgery.

### Bilateral Common Carotid Arteries Occlusion Surgery

In order to reduce animal mortality, we applied a modified bilateral ligation. In brief, the unilateral common carotid arteries of rats, anesthetized with isoflurane/air mixture (4 l/min for induction and 2 l/min for maintenance), were exposed and permanently ligated with 5-0 silk sutures *via* an incision on the same side, then the rats were returned to their own cages after recovering from anesthesia. Ligation of the carotid artery on the other side was performed in the same manner after two days. Rats in the Sham group were anesthetized, and the carotid artery was exposed, but without ligation. All rats were allowed to recover for 14 days after the first surgery.

### Chronic Restraint Stress

Chronic restraint stress (CRS) is always used to induce anxious and depressive symptoms associated with physiological changes. In this experiment, rats were exposed to CRS for a series of 14 consecutive days. In experiment one, the rats were randomly designated into four groups: Sham (without stress), CRS (Sham with CRS), Bilateral common carotid arteries occlusion (BCCAO) (without stress), and BCCAO/CRS (BCCAO with CRS). Rats which received BCCAO were randomly divided into two groups: BCCAO (without stress) and BCCAO/CRS (BCCAO with CRS). Rats were restrained in homemade restraint equipment for 6 h daily from 9:00 to 15:00 in the CRS group. Rats in the groups of Sham and BCCAO were kept in the cages without stress instead. In the experiment 2, the rats were randomly designated into three groups: Sham (without stress), BCCAO/CRS (BCCAO with CRS), and BCCAO/CRS + TMP (BCCAO with CRS and TMP administration).

### Drug Administration

Tetramethylpyrazine (TMP) was purchased from Sigma-Aldrich (St. Louis, MO, United States) and dissolved in 0.9% saline. TMP was intravenously infused through the tail vein at the dose of 30 mg/kg body weight daily. In the BCCAO/CRS + TMP group, rats received TMP from the first day of CRS once a day for 14 days 1h after restraint stress ended. Rats in groups of Sham and BCCAO/CRS received the same volume of 0.9% saline as TMP-treated rats.

### Body Weight and Food Intake Recording

The body weight of rats was measured and recorded every three days after surgery until the end of the experiment. At the same time, home-cage chow consumption over a 24 h period was measured. During this time, a pre-weighed food ration was provided in the morning (8:00 am) and daily food intake was measured by subtracting the amount of remaining food (including pieces inside the cage) from the pre-weighed food ration 24 h later.

### Behavioral Test

**FIGURE 1:**
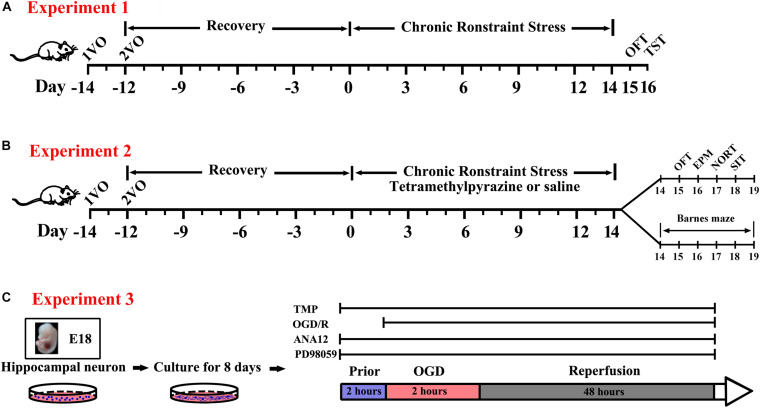
Experimental protocol for *in vivo* and *in vitro* experiments in the present study. **(A)** Open field test (OFT) and tail suspension test (TST) were used to test the efficiency of the establishment of the rat BPSD model using a bilateral internal carotid artery occlusion (BCCAO) method combined with chronic restraint stress (CRS) *in vivo* (Experiment 1). **(B)** Exploring the effect of Tetramethylpyrazine (TMP) on behavioral recovery using a battery of behavioral assays following BCCAO/CRS in the rat BPSD model *in vivo* (Experiment 2). **(C)** Uncovering the role of TrkB/ERK/CREB signaling pathway in TMP exerting neuroprotective effect through fortifying synaptic remolding *in vitro* experiments (Experiment 3).

For behavioral tests, all rats were performed according to the design in experiments one and two (Figures 1A,B). All experiments and analyses were conducted by individual investigators blinded to each designed group, and detailed procedures were described as follows.

For the Open Field Test (OFT), rats were introduced to the corner of an open arena (100 × 100 × 40 cm) and explored freely for 5 min. The arena was partitioned into 25 (5 × 5) equal-size squares and the middle nine grids (3 × 3) were considered as the central zone. Times spent in the center of the arena, moving distance, and speed were recorded by a camera. Then, the behavioral records were analyzed by AVTAS version 4.0 system (Yihong Technology, Wuhan, China).

For the Tail Suspension Test (TST), the tail of the rat was fixed on the crossbar which was about 1 m away from the ground. The immobile time of each rat was recorded within 5 min, including the period when the whole body was shaking, the limbs and head were relatively motionless. Rats were not allowed to climb up their tails and fall off the end of their tails during suspension during test.

For the Social Interaction Test (SIT), rats were adapted to the examining room for at least 1 h before the test. The test apparatus was a transparent Plexiglas box (60 W × 40 D × 20 H cm) divided into left, center, and right chambers with two transparent partitions with square opening doors (8 × 6 cm). For habituation, every rat was to allow to explore the entire environment for 10 min left the doors open. An empty cylindrical wire-cage (10 cm in diameter) was used as an inanimate object stimulus (chamber 3) and an identical cylindrical wire-cage containing a novel rat was used as the social stimulus (chamber 2). Each rat was placed in the center chamber with the doors closed. Once opening the doors, rat behavior was recorded using a video camera to capture the times and number of rats entries into chamber two and the first sniffing time to the rat in chamber two for 5 min to validate sociability.

For the Elevated Plus Maze (EPM), the instrument included two open and two closed arms at 50 × 10 cm each, and a 10 × 10 cm center area. Closed arms were enclosed with black walls measuring 50 cm in height. The maze was placed 60 cm from the floor. At the beginning, rats were placed in the center area facing the open arm. Then, time spent in the open arms, open-arm entries, and the stretching behaviors were measured by automatic animal behavior analysis software (Yihong Technology, Wuhan, China).

For the Novel Object Recognition Test (NORT), the experiment includes three phases: environment habituation, object familiarity period, and novel object recognition period. During the environment habituation, rats was randomly placed in an empty experimental box (50 × 50 × 40 cm) for 3 min. Next, two identical objects (A and B) were placed in the experiment box for object familiarization. Thereafter, the examined rat was placed on the centerline of the two objects. Then, exploration time for A and B was respectively recorded. After 1 hour, one of the objects A or B was replaced with a new object C, and the exploration time of rats for A or B and C was recorded within 5 min. Discrimination index was calculated as exploration of novel object minus exploration of familiar object/total exploration time × 100%.

For the Barnes Maze, rats conducted positioning escape training from day 15 to 19. During each training session, every rat was placed on the platform and covered with a black box for 15 s. If the rat found the target hole within 4min, it was allowed to stay in the hole for 30 s. If not, the rat was led into the target hole and stayed in it for 30 s. The latency to locate the escape tunnel, movement distance, and number of errors were recorded by a camera. The target box was removed, and rats were assigned to explore freely in the platform for 4 min on day 20. Subsequently, the residence time of the rats in the target quadrant and the number of times of searching for the hole of the element were recorded. The maze was thoroughly cleaned with 75% ethanol and dried with towels between trials to remove olfactory cues after each test.

### Primary Hippocampal Neurons Culture

A total of 10 embryonic day 18 SD rats were purchased from Animal Experimental Center of Hubei University of Chinese Medicine and used for isolating primary hippocampal neurons as previously described (Roppongi et al., 2017). In brief, the whole brains were collected and placed in a 60 mm Petri dish containing 2 mL Hank’s Balanced Salt Solution (HBSS) on ice. Then, the meninges were stripped away from the midline of the cerebral hemispheres under a dissecting microscope to dissect out the hippocampi. Subsequently, the obtained samples were digested with 0.15% trypsin at 37°C for 10 min after hippocampal tissues were transferred into a 15 mL tube. Afterward, samples were gently washed twice in Dulbecco’s modified Eagle’s medium (DMEM; Hyclone, Logan, Utah) supplemented with 10% fetal bovine serum (FBS, vol/vol, Hyclone, Logan, Utah) to inhibit the trypsin activity. Thereafter, the samples were triturated through a smooth-edged normal glass Pasteur pipette after being washed with DMEM. After the suspension settling for 5 min, the cell density from upper cell suspension was determined and seeded on poly-L-lysine (PLL; Sigma-Aldrich, Munich, Germany) coated dishes at a density of 1 × 10^5^/ml. Dissociated neurons were maintained with Neurobasal medium (Gibco, Grand Island, NY, United States) supplemented with 2% B27 (Gibco, Grand Island, NY, United States) and 1% L-Glutamine (200mM, Sigma-Aldrich, Munich, Germany). The culture medium was half-exchanged every 2–3 days and neurons were used for the present study from day 10 to 14 after being seeded on dishes. PD98059 (20 μM, cat. no. HY-12028, MedChemExpress LLC, Shanghai, China) and ANA-12 (10 μM, cat. no. HY-12497, MedChemExpress LLC, Shanghai, China) were prepared in DMSO in the stock solution (10 mM) and diluted in culture medium at the final concentration. For groups of Control and Oxygen glucose deprivation (OGD), the same amount of DMSO was added in the culture medium.

### Oxygen Glucose Deprivation and Reoxygenation

To mimic ischemia, neurons were subjected to OGD/reoxygenation (OGD/R) and the experimental procedures was shown in Figure 1C. In brief, culture plates were placed in an anaerobic chamber containing 5% CO_2_ and 95% N_2_ (< 1% O2) at 37°C after media were replaced by glucose-free HBSS. Two hours later, the OGD was terminated by bringing the plates back to a normoxic chamber after culture medium were exchanged to prewarmed Neurobasal medium supplemented with B27 and L-Glutamine as mentioned above for another 48 h. Neuron cultures were terminated to run tests after OGD procedures. To determine the effect of TMP on neurons, cultured neurons were pretreated with 100 μM TMP for 2 h prior to OGD and presented during the whole experiment of OGD/R. Cells in control group were treated identically except that they were not exposed to OGD.

### Western Blotting

The whole lysate was extracted from neurons or hippocampal tissues in different groups, and the concentration was determined by a BCA kit (Beyotime, Beijing, China) according to the manufacturer’s instruction. Then, samples were separated by electrophoresis in 8% or 10% SDS–PAGE, and transferred onto electro-blotted to polyvinylidene difluoride (PVDF) membranes (Millipore, Burlington, MA, United States). Subsequently, membranes were incubated in primary antibodies at 4°C overnight after they were blocked with 5% skim milk or 5% BSA at room temperature for 2 h. Thereafter, membranes were incubated in horseradish peroxidase (HRP)-conjugated secondary antibody at room temperature for 2 h after being rinsed twice with TBST. The signals for every band were detected using ECL luminous fluid (Beyotime, Beijing, China) in a ChemiDoc^TM^ XRS^+^ imaging system (Bio-Rad, CA, United States). Densitometric measurement of each membrane was performed using ImageJ. The primary antibodies used in the present study were as follows: TrkB (1:1000, cat. no. ab18987, Abcam, Cambridge, United Kingdom), p-TrkB (1:1000, cat. no. 4168S, Cell Signaling Technology, Danvers, MA, United States), ERK (1:1000, cat. no. 16443-1-AP, Proteintech Group, Inc., Wuhan, China), p-ERK (1:1000, cat. no. 4370S, Cell Signaling Technology, Danvers, MA, United States), CREB (1:1000, cat. no. 9197S, Cell Signaling Technology, Danvers, MA, United States), p-CREB (1:1000, cat. no. 9198S, Cell Signaling Technology, Danvers, MA, United States), PSD95 (1:1000, cat. no. 36233S, Cell Signaling Technology, Danvers, MA, United States), SYN (1:1000, cat. no. 5297S, Cell Signaling Technology, Danvers, MA, United States), SYP (1:1000, cat. no. 12270S, Cell Signaling Technology, Danvers, MA, United States), GAP43(1:1000, cat. no. 8945S, Cell Signaling Technology, Danvers, MA, United States), GAPDH (1:1000, cat. no. 60004-1-Ig, Proteintech Group, Inc., Wuhan, China), and Actin (1:1000, cat. no. 66009-1-Ig, Proteintech Group, Inc., Wuhan, China).

### Golgi-Cox Staining

The brain right hemispheres were collected for Golgi-cox staining using a rapid Hito Golgi-Cox OptimStain^TM^ Prekit (cat. no. HTKNS1125NH, Hitobiotec Corp., Kingsport, TN, United States) according to the manufacturer’s instructions. Briefly, fresh brain was collected and rinsed with normal saline and then immersed in the mixture solution of 1:1 volumetric ratio of Hito impregnation solution mixture of A: B for 14 days at room temperature in the dark (replaced once after 24 h of immersion). After impregnation, brains were transferred into solution-3 and stored in the dark for 2 days at 4°C (the solution was replaced once after 12 h). Afterward, the brain sample was mounted on vibrating slicer fixed table and sectioned at 80 μm. The brain sections were mounted on a glass slide coated with chromic acid gelatin, then stained and further processed according to the manufacturer’s instructions and sealed with resinous mounting medium (cat. no. 10004160, Sinopharm Chemical Reagent Co., Ltd., Shanghai, China). Finally, slices were observed and captured using a light microscope (BX63, Olympus, Japan).

### Dendritic Spine Analysis

For dendritic spine analysis, nine sections were analyzed, and the cross-sectional areas were calculated and reported as the average of four independent measurements. Images from CA1 pyramidal neurons and DG of the hippocampus were captured by a light microscope (BX63, Olympus, Japan), and investigators were blind to each sample. Then, the images were analyzed using Image J software (NIH, Bethesda, MD, United States). A part of complete dendrites was selected as the analysis object in each section, and the selected dendrites were separated from other organizational structures so as not to confuse the judgment of the software. Secondary dendrites and beyond were analyzed and at least three dendritic segments longer than 10 μm were analyzed in each neuron. The measurements for basal and apical dendrites were performed separately. Subsequent analysis of these images was carried out on the software Reconstruct^1^. Length of each imaged dendritic segment was measured through Z trace and corresponding spines were identified and counted by three independent investigators in a double-blind manner. For each neuron, the final spine densities were averaged from results obtained by three investigators. Sholl analysis was used to measure dendritic length and number of spines within concentric circles at increasing diameters of 10 μm steps from reconstructed neurons.

### Transmission Electron Microscopy

Transmission electron microscopy (TEM) was carried out as previously described (Jiang et al., 2020). The rat hippocampal tissues were soaked in 2.5% glutaraldehyde for at least 4 h at 4°C, and then transferred to 1% citric acid for fixation. Then, they were dehydrated with gradient acetone after soaking in uranyl acetate. Thereafter, samples were embedded with epoxy resin and sliced into 70–90 nm. They were counterstained with lead citrate after placing on the copper trough grid and the ultrastructure of nerve tissue was observed under transmission electron microscope Tecnai G220 (FEI, Hillsboro, OR, United States). Approximately 10–15 pictures were captured for each group of 3 different samples. All synapses were analyzed and counted by two experimenters blind to sample information.

### Immunofluorescence

For immunofluorescence, neuron coverslips or frozen brain sections (−20°C) from each group were post-fixed using 4% paraformaldehyde (PFA) in 0.01 M phosphate buffer saline (PBS) for 1 h at room temperature after being rinsed twice with PBS. Then, the samples were blocked with 5% bovine serum album (BSA; Beyotime, Beijing, China) supplemented with 0.3% Triton X-100 (Sigma-Aldrich, Munich, Germany) in PBS for 2 h at room temperature. Afterward, samples were incubated with CREB (1:100, cat. no. 9197S, Cell Signaling Technology, Danvers, MA, United States), p-CREB (1:100, cat. no. 9198S, Cell Signaling Technology, Danvers, MA, United States), SYN (1:100, cat. no. 5297S, Cell Signaling Technology, Danvers, MA, United States), and MAP2 (1:100, cat. no. 67015-1-Ig, Proteintech Group, Inc., Wuhan, China) overnight at 4°C. Next, samples were incubated with Alexa Fluor^®^ 555 or 488-conjugated secondary antibody (1:300; cat. nos. A0453 and A0423; Beyotime, Beijing, China) at room temperature for 2 h. Cell nuclei were counterstained with 4′,6-diamidino-2-phenylindole (DAPI, Sigma-Aldrich, Munich, Germany) for 10 min at room temperature. Subsequently, samples were mounted onto glass slides and images were captured using a confocal microscope (LSM780; Carl Zeiss, Weimar, Germany). Meanwhile, blocking buffer without the primary antibodies was used as the negative control. Image-Pro Plus 6.0 software (Media Cybernetics, Inc., Rockville, MD, United States) was used for quantitative analysis of fluorescence microscopy images. For each sample, at least six fields were captured, analyzed, and the cross-sectional areas were calculated and reported as the average of four independent measurements. All measurements were performed by an individual investigator who was blinded to the experiment groups.

### Statistical Analysis

All data were presented as the mean ± SE, and analyzed using GraphPad Prism 6.0 software (GraphPad Software, Inc., San Diego, CA, United States) or SPSS 19.0 software (SPSS Inc., Chicago, IL, United States). Comparisons between two groups were performed using Student’s t tests or analysis of variance (ANOVA), followed by Tukey’s *post hoc* test if the data exhibited a normal distribution using a Shapiro–Wilk normality test. The standard error (SE) of the mean normalized to negative control was determined using the Delta method (Polli et al., 1997), Independent-sample T test or single-sample *T*-test were used to compare the data after Delta adjustment. Continuous measurements were analyzed using repeated ANOVA, followed by Bonferroni *post hoc* tests. A *P* < 0.05 value was considered as a significant difference.

## Results

### Establishment of BPSD Model Using Bilateral Internal Carotid Artery Occlusion Method Combined With Chronic Restraint Stress Displayed Severe Anxiety and Depression-Like Behavior in Rats

**FIGURE 2:**
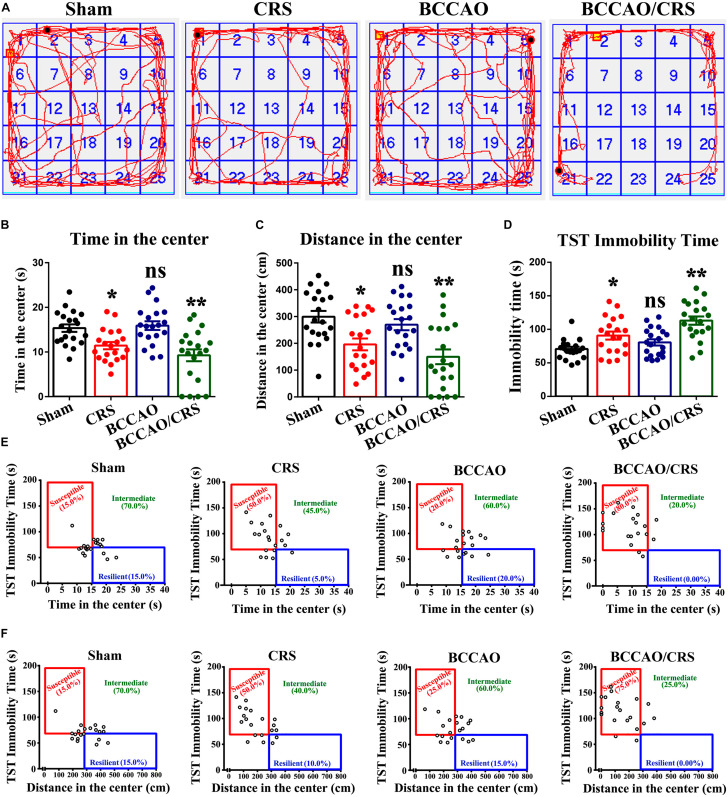
Behavioral tests validate successful establishment of rat BPSD model with severe depression and anxiety. **(A)** Representational trajectories of rats moving in the open field in different groups. **(B)** Bar summarizing the total time of rats in the center during open field test (OFT). **(C)** Quantitation of the total distance of rats in the center during open field test (OFT). **(D)** Statistical analysis of immobility time of rats in the tail suspension test (TST). **(E)** The total time in the center during OFT against immobility time during TST indicating the susceptibility to depressive and anxious phenotype in various groups. A median split on both times spent in the center area (median = 15 s) and immobility time (median = 70 s) was used to separate rats into susceptible, intermediate and resilient phenotypes in different groups. **(F)** The distance in the center during OFT against immobility time during TST delineating the susceptibility to depressive and anxious phenotype in various groups. A median split of distance (median = 280 cm) in the center using OFT and immobility time (median = 70 s) using TST was performed to separate rats into susceptible, intermediate and resilient phenotypes in different groups. *P < 0.05, **P < 0.01, ^ns^*P* > 0.05 vs. Sham. Data are presented as mean ± SE. *n* = 20 in each group.

To face the dilemma of the prevalence of BPSD in the neurodegenerative disorders, there is an urgent need to develop more translatable animal models with similarities to humans in both the symptomatology and physiopathology of dementia. Here, we performed permanent occlusion of the bilateral common carotid arteries (2-vessel occlusion, 2VO) to simulate the cerebral hypoperfusion condition in neurodegenerative processes, combined with chronic restraint stress (CRS) in rats. As shown in Figure 1A, rats received BCCAO surgery followed by CRS for a consecutive 14 days. Then, an open field test (OFT), one of the most popular ethological tests to assess anxiety-like behavior in rodents (Kuniishi et al., 2017), was used to evaluate the severity of anxiety in rats among different groups. The results indicated that the total time and distance rats spent in the center exhibited a visible decrease in BCCAO/CRS group, compared to sham groups (Figures 2A,B, *F* (1, 76) = 27.760; *P* = 0.0003; Figures 2A–C, *F* (1, 76) = 23.190; *P* = 0.0001). Meanwhile, rats represented anxiety, in some degree, in the group of CRS (Figures 2A,B, *F* (1, 76) = 27.760; *P* = 0.0428; Figures 2A–C, *F* (1, 76) = 23.190; *P* = 0.0141). Interestingly, rats showed no obvious difference between the groups of BCCAO and Sham (Figures 2A,B, *F* (1,76) = 0.639; *P* > 0.05; Figures 2A–C, *F* (1, 76) = 2.695; *P* > 0.05). Furthermore, a Tail Suspension Test (TST), one of the most common tests to examine depression-like behavior (Arauchi et al., 2018; Zong et al., 2018), was conducted to assess the degree of depression in rats among different groups. Our data demonstrated that rats represented the longest immobility time in group BCCAO/CRS, in comparison with sham groups (Figure 2D, *F* (1, 76) = 25.750; *P* < 0.0001). Meanwhile, rats in the CRS group presented longer immobility time than that in the Sham group (Figure 2D, *F* (1, 76) = 25.750; *P* = 0.0459). In addition, the data collected from the two tests were integrated to tell the susceptibility of rats transforming to depressive and anxious phenotypes. The integrated data revealed that rats showed the highest susceptibility transforming to depressive and anxious phenotypes in the BCCAO/CRS group, and higher susceptivity in CRS group, compared with the Sham and BCCAO groups (Figures 2E,F). Together, these results demonstrated that the strategy of establishing a rat BPSD model in the present study was a simple, practicable and time-saving approach, which could mimic the behavioral and psychological symptoms in rats with neurodegenerative diseases to some extent. Herein, we used this model in our future research to determine the effect of TMP on depressive and anxious behaviors in rat models, and we named it as the BPSD model.

### TMP Administration Significantly Attenuated Anxiety in Rats Resulting From BCCAO/CRS

**FIGURE 3:**
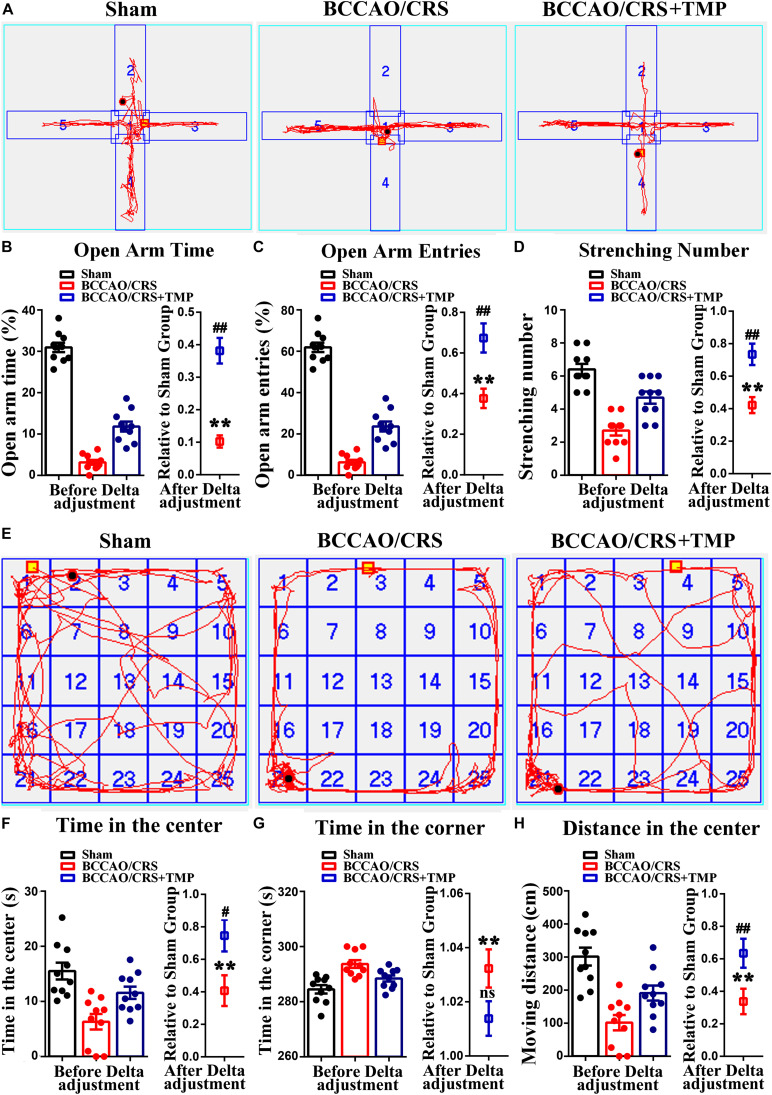
Tetramethylpyrazine (TMP) administration alleviates depression and anxiety in rats caused by BCCAO/CRS. **(A)** Representational traces showing rats moving in the elevated plus maze (EPM) test. **(B)** Bargraph indicating the percentage of time that rats were moving/staying in the open arms. **(C)** Barchart demonstrating times that rats entered the open arms during EPM test. **(D)** Quantitation of the stretching number in rats during EPM test. **(E)** Typical route representing rats moving in the open field. **(F)** Summarized data demonstrating the total time of rats in the center during OFT. **(G)** Quantitative data summarizing the total time of rats in the corner during OFT. **(H)** Quantitative analysis of rats moving distance during the OFT. **P < 0.01 vs. Sham. ^#^*P* < 0.05, ^##^*P* < 0.01, ^ns^*P* > 0.05 vs. BCCAO/CRS. Data are presented as mean ± SE. *n* = 10 in each group.

Considering that TMP hold pleiotropic neuroprotective effects, we evaluated the role of TMP in treating anxious behavior based on BPSD model using elevated plus maze (EPM) test. Firstly, the effect of TMP on weight gaining and food intake was calculated. The quantitative data represented that there were no predominant differences among the three groups (Supplementary Figure 1A, *F* (1, 9) = 1.781; *P* = 0.2150; Supplementary Figure 1B, *F* (1, 9) = 2.826; *P* = 0.1270). Subsequently, the results presented that TMP administration evidently increased the percentage of time that rats were moving or staying in the open arms (Figures 3A,B, *t* (18) = 6.416; *P* < 0.0001), and the times taken by rats to enter the open arms (Figures 3A,C, *t* (18) = 3.454; *P* = 0.0028), as well as the stretching number in rats during EPM test (Figures 3A,D, *t* (18) = 3.803; *P* = 0.0013). Besides, the OPT was further performed to certify the effect of TMP on anxious behavior improvement. The results exhibited that TMP administration dramatically augmented the total time (Figures 3E,F, *t* (18) = 2.497; *P* = 0.0224) and distance (Figures 3E,H, *t* (18) = 2.499; *P* = 0.0224) rats spent in the center in group BCCAO/CRS + TMP, compared to BCCAO/CRS. However, the total time of rats in the corner showed similar differences between the group of BCCAO/CRS and BCCAO/CRS + TMP (Figures 3E,G, *t* (18) = 1.944; *P* = 0.0677). Together, these data indicated that TMP application dramatically attenuated anxiety in rats induced by BCCAO/CRS.

### TMP Application Clearly Ameliorated Recognitive Ability and Sociability in BCCAO/CRS Rats

**FIGURE 4:**
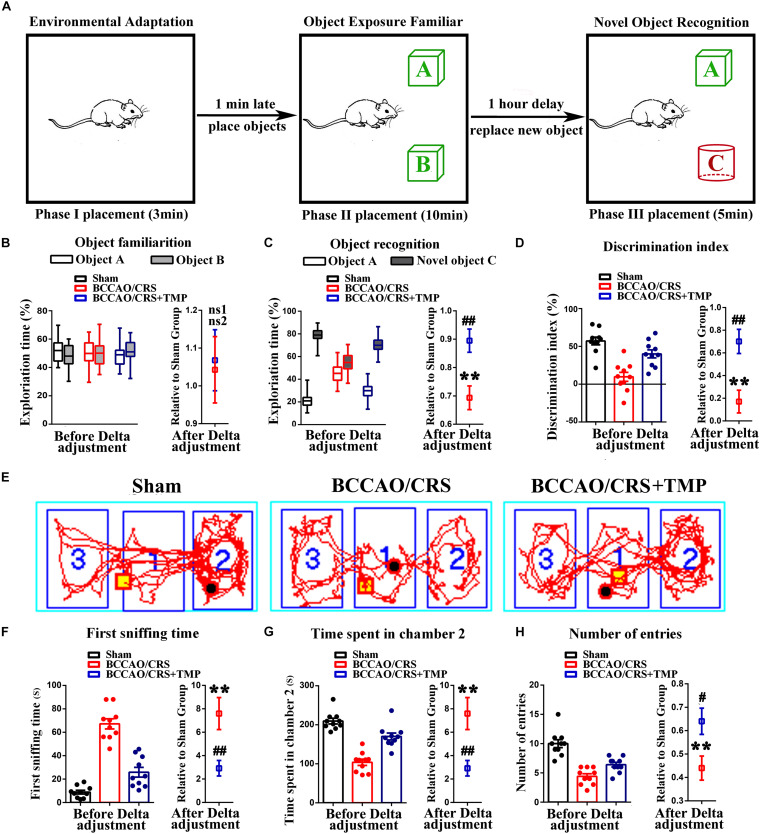
TMP improves recognitive ability and sociability in BCCAO/CRS rats. **(A)** Diagrammatic sketch of novel object recognition test (NORT). **(B)** Time of rats spent on exploring two identical object A and B during the period of familiarization. **(C)** Time of rats spent on exploring old object A or B and novel object C during the period of recognition. **(D)** Novel object discrimination index of rats during the period of recognition. **(E)** Representative traces of rats moving in the 3-chamber sociability test. **(F)** Time that rats exerting first sniffing at additional rats in the social interaction test (SIT). **(G)** Bargraph indicating the time of social interaction during SIT. **(H)** Times that rats entered the chamber 2 during SIT. **P < 0.01, ^ns1^*P* > 0.05 vs. Sham. ^#^*P* < 0.05, ^##^*P* < 0.01, ^ns^*P* > 0.05 vs. BCCAO/CRS. Data are presented as mean ± SE. *n* = 10 in each group.

Given the multiple psychological behaviors of BPSD, we next conducted a novel object recognition test (NORT) and social interaction test (SIT) to estimate the recognitive ability and sociability in rats from different groups. The results from the NORT showed that there were no significant differences in exploration time between object A and B among the three groups in the object familiarization period (Figures 4A,B, *t* (18) = 0.2127; *P* = 0.8339). However, the percentage of exploration time represented visible differences between object A or B (familiar object) and the novel object C in group of Sham and BCCAO/CRS + TMP with a longer exploration time for the novel object than the BCCAO/CRS group (Figures 4A,C, *t* (18) = 3.442; *P* = 0.0029). Similarly, the discrimination index in BCCAO/CRS group was lower than that of the Sham and BCCAO/CRS + TMP groups during the recognition phase (Figure 4D, *t* (18) = 3.623; *P* = 0.0019). Thereafter, another SIT was conducted to distinguish the social behavior of rats. The results revealed that the first sniffing time in BCCAO/CRS was longer than the Sham group (Figures 4E,F, *t* (9) = 4.831; *P* = 0.0009), while TMP administration could have partially abrogated this inhibitory effect (Figures 4E,F, *t* (18) = 3.078; *P* = 0.0065). In contrast, time spent by rats in chamber 2 was obviously decreased in rats with BCCAO/CRS(Figures 4E,G, *t* (9) = 12.740; *P* < 0.0001), and TMP could enhance the duration of rats spent in chamber 2 (Figures 4E,G, *t* (18) = 5.030; *P* < 0.0001). Coincidently, rats in the BCCAO/CRS + TMP group exhibited a prominently elevated number of entries to the chamber 2, compared with that in the BCCAO/CRS group (Figures 4E,H, *t* (18) = 2.625; *P* = 0.0172). These results assured that TMP improved recognitive ability and sociability in BCCAO/CRS rats to some degree.

### TMP Attenuated Learning and Memory Impairments in BCCAO/CRS Rats

**FIGURE 5:**
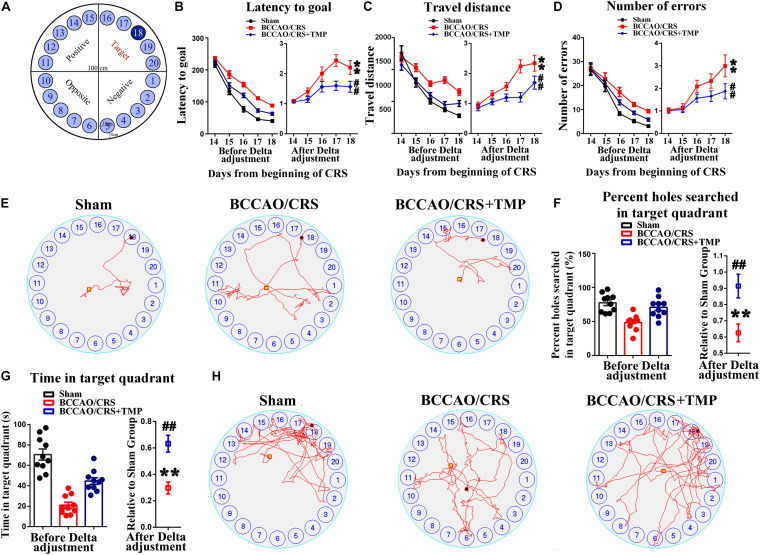
TMP ameliorates learning and memory deficits in BCCAO/CRS rats. **(A)** The diagrammatic sketch of the zone and magnitude of Barnes maze instrument. **(B)** Chart showing the average latency of rats finding out the escape tunnel in different groups from day 15 to 19. **(C)** The average traveled distance of rats finding out the escape tunnel in different group from day15 to 19. **(D)** Total number of errors during experiments in different groups from day 15 to 19. **(E)** Representative traces of rats finding out the escape tunnel on day 19 in various groups. **(F)** Percentage holes searched in the target quadrant during the probe trial. **(G)** Total time of rats spent on exploring the target quadrant during the probe trial. **(H)** Representative trajectories in the quadrant zone during spatial memory probe trial. ^∗∗^*P* < 0.01 vs. Sham. ^##^*P* < 0.01 vs. BCCAO/CRS. Data are presented as mean ± SE. *n* = 10 in each group.

Chronic cerebral hypoperfusion always exerts damage to the hippocampus, which is associated with learning and memory deficits (Zhang et al., 2017; Mansour et al., 2018; He et al., 2019). Hence, we therefore used the Barnes maze paradigm to evaluate hippocampus-dependent spatial memory and learning with addition of TMP (Figure 5A). Our results presented that the escape latency of rats was obviously decreased in the BCCAO/CRS + TMP group compared to that in the BCCAO/CRS group (Figure 5B, *F*_day__18_ (1, 9) = 14.992; *P* = 0.0040). Analogously, the travel distance and searching errors of rats in the BCCAO/CRS + TMP group to find the escape tunnel were significantly less than that of rats in BCCAO/CRS group (Figure 5C, *F*_day__18_ (1, 9) = 5.421; *P* = 0.0450; Figure 5D, *F*_day__18_ (1, 9) = 30.205; *P* < 0.0001). Moreover, the representative trajectories showed that the rats in BCCAO/CRS + TMP group were easier to find the escape tunnel than that in BCCAO/CRS group on day 18 (Figure 5E). Similarly, the percent and frequency of searching holes in the target quadrant for rats in the BCCAO/CRS + TMP group were evidently raised than that in the BCCAO/CRS group (Figure 5F, *t* (18) = 3.170; *P* = 0.0053; Figure 5G, *t* (18) = 4.292; P=0.0044). In addition, the typical traces intuitively displayed that rats in the BCCAO/CRS + TMP group held better spatial memory than that in the BCCAO/CRS group during the test period on day 18 (Figure 5H).

### TMP Alleviated Dendritic and Spine Deficits and Upregulated the Expression of Synapse-Related Proteins in Hippocampus in BCCAO/CRS Rats

**FIGURE 6:**
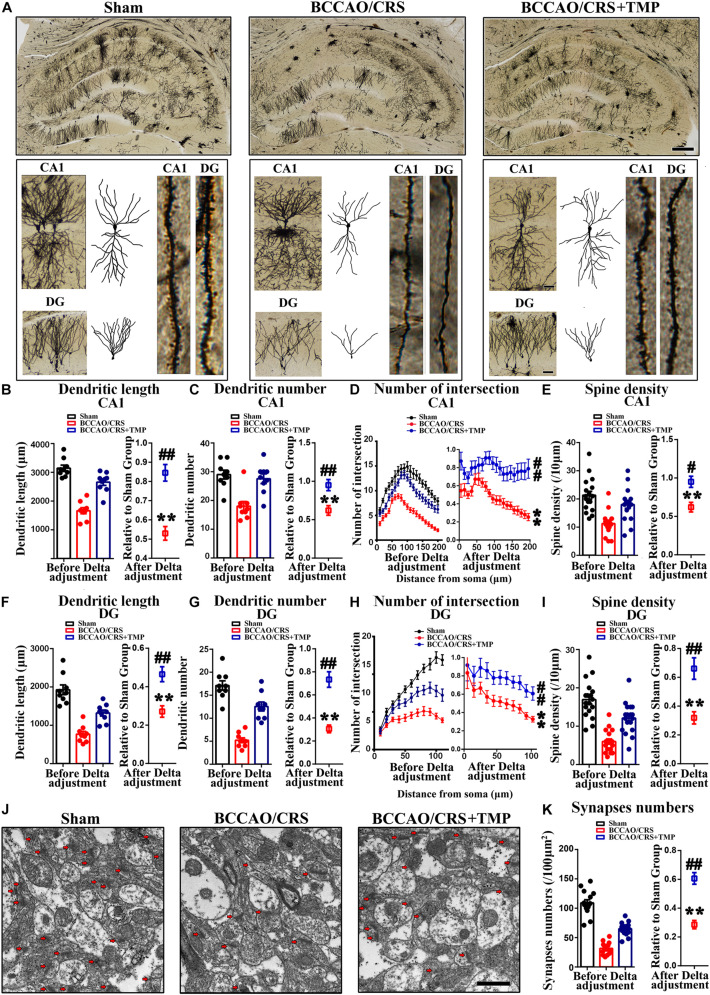
TMP attenuates dendritic and spine deficits in hippocampus in BCCAO/CRS rats. **(A)** Representative Golgi-Cox staining images exhibiting dendritic arborization in hippocampal CA1 pyramidal neurons and DG granular neurons in hippocampal slices. Scale bar: 400 μm; and 50 μm for enlarged inserts. **(B)** Quantification of the length of dendrites in hippocampal CA1 pyramidal neurons. **(C)** Bar chart indicating the number of dendrites in hippocampal CA1 pyramidal neurons. **(D)** Quantitative analysis of the number of intersections of dendrites in hippocampal CA1 pyramidal neurons. **(E)** Spine density of dendrites in hippocampal CA1 pyramidal neurons. **(F)** Quantification of the length of dendrites in hippocampal DG granular neurons. **(G)** Quantification of the number of dendrites in hippocampal DG granular neurons. **(H)** Chart indicating the number of intersections of dendrites in DG granular neurons. **(I)** Spine density of dendrites in hippocampal DG granular neurons. **(J)** Representative images of hippocampal synapses under transmission electron microscope (TEM). Scale bar: 1 μm. **(K)** Quantitative statistics of hippocampal synapses in different groups. **P < 0.01 vs. Sham. ^#^*P* < 0.05, ^##^*P* < 0.01 vs. BCCAO/CRS. Data are presented as mean ± SE. *n* = 3 in each group, and 3 slices per rat for Golgi-Cox staining, 5 random fields per rat for TEM.

In order to explore the underlying mechanism responsible for the observed improvement in behavior and psychological symptoms, we explored alterations in dendritic spine density and morphology. Golgi-Cox staining images showed that the dendritic length (Figures 6A,B), dendritic number (Figures 6A–C), and branches (Figures 6A–D) in CA1 from BCCAO/CRS rats were significantly less than that in Sham rats (Figures 6A,B, *t* (8) = 13.270; *P* < 0.0001; Figures 6A–C, *t* (8) = 6.113; *P* = 0.0003; Figures 6A–D, *F* (1, 320) = 804.400; *P* < 0.0001), whereas TMP reversed the reduction of dendritic branches induced by BCCAO/CRS (Figures 6A,B, *t* (16) = 5.635; *P* < 0.0001; Figures 6A–C, *t* (16) = 3.449; *P* = 0.0033; Figures 6A–D, *F* (1, 320) = 240.400; *P* < 0.0001). At the same time, the spine density in CA1 from the BCCAO/CRS rats were significantly less than that in Sham rats (Figures 6A–E, *t* (17) = 7.743; *P* < 0.0001), whereas TMP reversed the reduction of dendritic branches induced by BCCAO/CRS (Figures 6A–E, *t* (34) = 2.508; *P* = 0.0171). Analogously, the dendritic length (Figures 6A–F), dendritic number (Figures 6A–G), and branches (Figures 6A–H) in DG were more in the BCCAO/CRS + TMP group than that in the BCCAO/CRS group (Figures 6A–F, *t* (16) = 3.973; *P* = 0.0011; Figures 6A–G, *t* (16) = 5.679; *P* < 0.0001; Figures 6A–H, *F* (1, 176) = 44.680; *P* < 0.0001). The spine density in DG exhibited the same trend as that in CA1 (Figure 6A–I, *t* (34) = 3.993; *P* = 0.0003). Subsequently, the representative transmission electron microscopy images illustrated that the number of synapses (100 μm^2^ for counting) was highly abundant in the Sham group, and BCCAO/CRS evidently reduced the number of synapses (Figures 6J,K, *t* (14) = 24.730; *P* < 0.0001), while TMP rescued the loss of synapses in BCCAO/CRS rats (Figures 6J,K, *t* (28) = 6.488; *P* < 0.0001).

**FIGURE 7:**
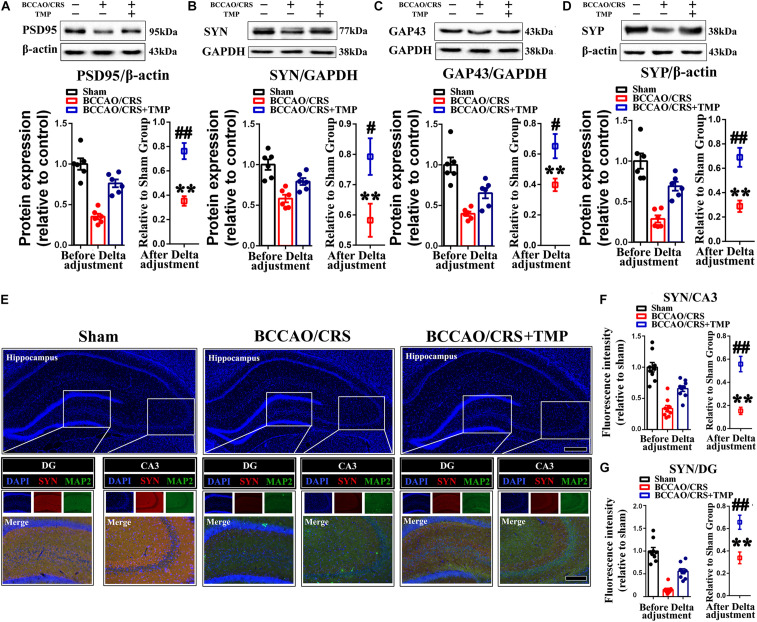
TMP increases synapse-associated protein expression in BCCAO/CRS rats. **(A–D)** Representative immunoblots representing the expression and semi-quantitative analysis of synapse-associated proteins including PSD-95 **(A)**, SYN **(B)**, GAP-43 **(C)**, and SYP **(D)**. **(E)** Representative immunostaining showing the colocalization of MAP (green) and SYN (red) in the hippocampus of CA3 and DG. The inserts were high magnification from each group. Nuclei were counterstained with DAPI. Scale bar: Scale bar: 400 μm; and 200 μm for enlarged inserts. **(F)** Quantification of the fluorescence intensity of SYN in hippocampal CA3. **(G)** Bar graph summarizing the fluorescence intensity of SYN in hippocampal DG. **P < 0.01 vs. Sham. ^#^*P* < 0.05, ^##^*P* < 0.01 vs. BCCAO/CRS. Data are presented as mean ± SE. n = 6 per group for Western blotting; *n* = 3 in each group and 3 slices per rat for immunofluorescence.

Furthermore, the expression of synaptic markers was evaluated in the hippocampus. The bands illustrated that the expression of PSD95 (Figure 7A), SYN (Figure 7B), GAP43 (Figure 7C), and SYP (Figure 7D) was substantially decreased in the BCCAO/CRS group(Figure 7A, *t* (5) = 16.430; *P* < 0.0001; Figure 7B, *t* (5) = 7.649; *P* = 0.0006; Figure 7C, *t* (5) = 14.520; *P* < 0.0001; Figure 7D, *t* (5) = 15.150; *P* < 0.0001), while this effect could be partially abolished after TMP administration (Figure 7A, *t* (10) = 5.309; *P* = 0.0003; Figure 7B, *t* (10) = 2.578; *P* = 0.0275; Figure 7C, *t* (10) = 2.831; *P* = 0.0178; Figure 7D, *t* (10) = 4.409; *P* = 0.0013). Meanwhile, the colocalization of MAP2 with SYN using immunostaining was conducted to echo the tendency obtained from western blot. The results demonstrated that the abundance of SYN was markedly decreased at CA1 and DG in the BCCAO/CRS group (Figures 7E,F, *t* (8) = 12.630; *P* < 0.0001; Figures 7E,G, t (5) = 27.270; P < 0.0001), and this inhibitory effect was evidently abrogated with TMP treatment (Figures 7E,F, *t* (16) = 3.888; *P* = 0.0013; Figures 7E–G, *t* (16) = 5.520; *P* < 0.0001). Collectively, our results uncovered the reason why TMP ignited pleiotropic neuroprotective effects on BPSD was by virtue of modulating synaptic remodeling in hippocampus in BCCAO/CRS rats.

### TMP Activated TrkB/ERK/CREB Signaling Pathway to Exert a Neuroprotective Effect *via* Fortifying Synaptic Remolding *in vivo* and *in vitro*

**FIGURE 8:**
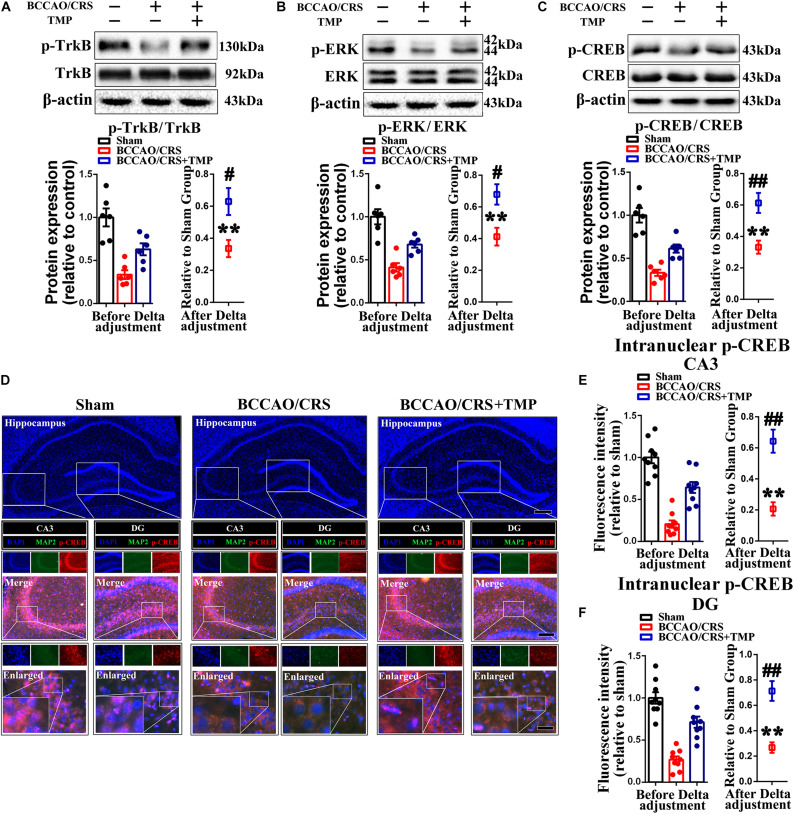
TMP activates TrkB/ERK/CREB signaling pathway to exert neuroprotective effect in BCCAO/CRS rats. **(A–C)** Typical immunoblots exhibiting the expression and semi-quantitative analysis of proteins related to TrkB/ERK/CREB signaling pathway including p-TrkB **(A)**, p-ERK **(B)** and p-CREB **(C)**. **(D)** Representative immunostaining depicting the colocalization of MAP2 (green) and CREB (red) in hippocampus of CA3 and DG. The inserts were high magnification from each group. Nuclei were counterstained with DAPI. Scale bar: Scale bar: 400 μm; 200 μm for medial inserts, and 50 μm for enlarged inserts. **(E)** Quantification of the optic intensity of intranuclear CREB in hippocampal CA3and DG. **(F)** Bar chart summarizing the optic intensity of intranuclear p-CREB in hippocampal CA3 and DG. **P < 0.01 vs. Sham. ^#^*P* < 0.05, ^##^*P* < 0.01 vs. BCCAO/CRS. Data are presented as mean ± SE. *n* = 6 per group for Western blotting; *n* = 3 in each group and 3 slices per rat for immunofluorescence.

Previous research has certified that TMP up-regulates the expression level of brain-derived neurotrophic factor (BDNF) to mediate neurogenesis (Wu et al., 2019) and local immunomodulation (Wu et al., 2020), implying that its co-effectors might be involved in TMP enhancing synaptic reestablishment. To test our hypothesis, we first evaluated the expression of coactivators (p-TrkB, p-ERK and p-CREB) using western blot. The bands depicted that the expression of p-TrkB, p-ERK and p-CREB was remarkably down-regulated in the hippocampus of rats in the BCCAO/CRS group (Figure 8A, *t* (5) = 12.420; *P* < 0.0001; Figure 8B, *t* (5) = 10.470; *P* = 0.0001; Figure 8C, *t* (5) = 16.000; *P* < 0.0001), while this phenomenon was reversed with administration of TMP to some degree (Figure 8A, *t* (10) = 2.94; *P* = 0.0148; Figure 8B, *t* (10) = 3.119; *P* = 0.0109; Figure 8C, *t* (10) = 3.699; *P* = 0.0041). Next, the dual immunostaining of MAP2 and p-CREB-the down-stream of TrkB/ERK/CREB signaling pathway delineated that the optic density of intranuclear p-CREB was visibly raised in the hippocampus of rats in the BCCAO/CRS + TMP group, in comparison with that in the BCCAO/CRS group (Figures 8D,E, *t* (16) = 5.084; *P* < 0.0001; Figures 8D–F, *t* (16) = 5.059; *P* = 0.0001).

**FIGURE 9:**
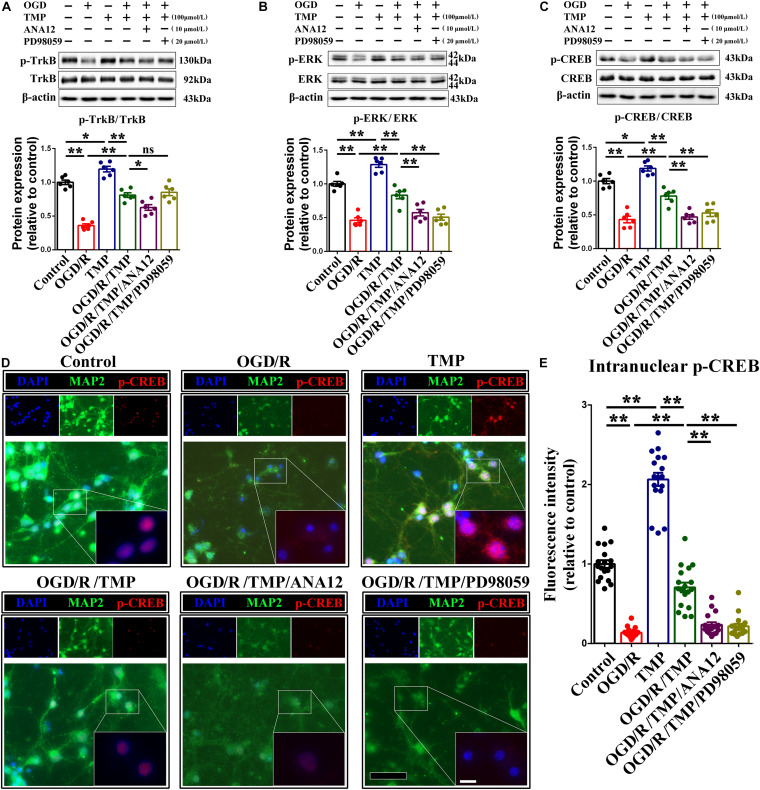
Activation of TrkB/ERK/CREB signaling pathway protects neurons against injury induced by oxygen glucose deprivation (OGD) condition. **(A–C)** Representative immunoblots representing the expression and semi-quantitative analysis of proteins related to TrkB/ERK/CREB signaling pathway including p-TrkB **(A)**, p-ERK **(B)** and p-CREB **(C)** in different groups. **(D)** Representative immunostaining exhibiting the colocalization of MAP2 (green) and p-CREB (red) in each group. Nuclei were counterstained with DAPI. Scale bar: Scale bar: 50 μm; and 20 μm for enlarged inserts. **(E)** Quantification of optic intensity of intranuclear p-CREB in various groups. Data are presented as mean ± SE. *n* = 6 per group, *P < 0.05, **P < 0.01.

To further determine the involvement of the TrkB/ERK/CREB signaling pathway in protecting neurons against chronic progressive ischemia in hippocampus, hippocampal neurons were cultured, as well as two specific inhibitors which were administrated with or without oxygen glucose deprivation (OGD) model, which is extensively used to simulate ischemia *in vivo* (Yang et al., 2019). Our results demonstrated that TMP substantially upregulated p-TrkB (Figure 9A), p-ERK (Figure 9B), and p-CREB (Figure 9C) expression, whose expressions were decreased with OGD, while this enhanced effect induced by TMP (Figure 9A, *F* (1, 20) = 90.590; *P* < 0.0001; Figure 9B, *F* (1, 20) = 59.570; *P* < 0.0001; Figure 9C, *F* (1, 20) = 38.330; *P* < 0.0001) was partially counteracted with addition of ANA12, a potent and selective TrkB antagonist (Ding et al., 2020), and/or PD98059, a ERK1/2 signaling inhibitor (Gao et al., 2018) (Figure 9A, F (2, 15) = 8.056; P = 0.0042; Figure 9B, *F* (2, 15) = 12.550; *P* = 0.0006; Figure 9C, *F* (2, 15) = 13.780; *P* = 0.0004), in primary hippocampal neurons. Meanwhile, immunostaining images attested that the optic density of p-CREB in nuclear was brighter with administration of TMP, while the density was the darkest in group OGD (Figures 9D,E, *F* (1, 68) = 201.8; *P* < 0.0001). This enhanced effect resulting from TMP was greatly abolished with ANA12 and/or PD98059 addition (Figures 9D,E, *F* (2, 51) = 40.92; *P* < 0.0001).

**FIGURE 10:**
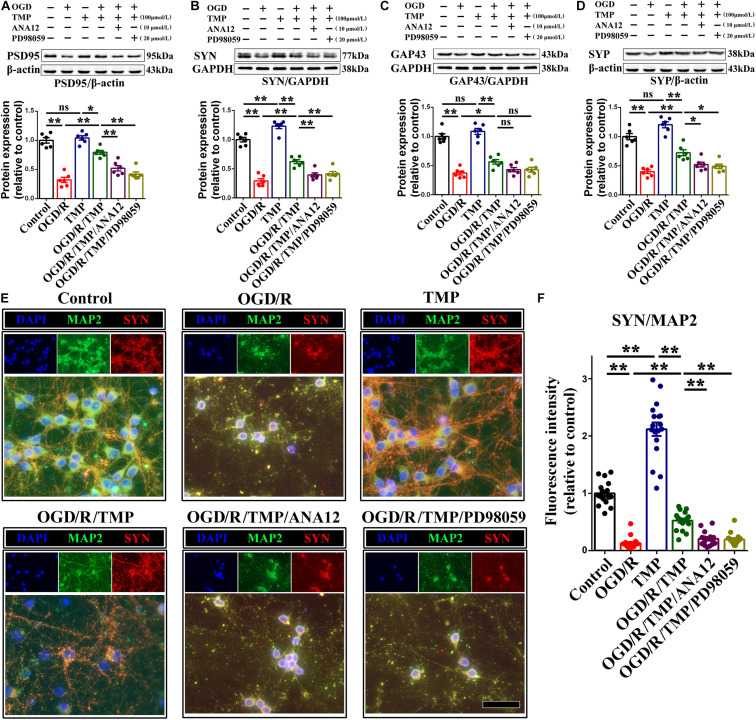
TMP administration increases synapse-associated protein expression in primary cultured neurons under OGD condition. **(A–D)** Representative immunoblots representing the expression and semi-quantitative analysis of synapse-associated proteins including PSD-95 **(A)**, SYN **(B)**, GAP-43 **(C)**, and SYP (D). **(E)** Representational immunostaining of co-labeling of MAP2 (green) and SYN (red) in primary neurons under different groups. Nuclei were counterstained with DAPI. Scale bar: 50 μm. **(F)** Bar graph illustrating the optic intensity of SYN in different groups. Data are presented as mean ± SE. *n* = 6 per group, *P < 0.05, **P < 0.01.

Additionally, for the effect of the TrkB/ERK/CREB signaling pathway on protecting synapses against hypoxia-induced injury in primary hippocampal neurons, we firstly examined the expression of synapse-related makers (including PSD95, SYN, GAP43 and SYP) under OGD condition. Our results demonstrated that TMP markedly increased the expression of PSD95 (Figure 10A), SYN (Figure 10B), GAP43 (Figure 10C), and SYP (Figure 10D), whose expression was substantially declined with OGD, while this enhanced effect generated from TMP (Figure 10A, *F* (1, 20) = 39.550; *P* < 0.0001; Figure 10B, *F* (1, 20) = 53.420; *P* < 0.0001; Figure 10C, *F* (1, 20) = 10.100; *P* = 0.0047; Figure 10D, *F* (1, 20) = 30.06; *P* < 0.0001)was abrogated with addition of ANA12 and/or PD98059, to some degree, in primary hippocampal neurons (Figure 10A, *F* (2, 15) = 23.410; *P* < 0.0001; Figure 10B, *F* (2, 15) = 12.810; *P* = 0.0006; Figure 10C, *F* (2, 15) = 3.938; *P* = 0.0422; Figure 10D, *F* (2, 15) = 8.057; *P* = 0.0042). Moreover, immunostaining pictures substantiated that the intranuclear optic density of p-CREB was higher with addition of TMP, compared to OGD group (Figures 10E,F, *F* (1, 68) = 127.600; *P* < 0.0001). However, this phenomenon triggered by TMP was neutralized with ANA12 and/or PD98059 addition, to some extent (Figures 10E,F, *F* (2, 51) = 39.730; *P* < 0.0001). Mechanically, these results proved that TMP could activate the TrkB/ERK/CREB signaling pathway to reestablish synapses *in vivo* and *in vitro*.

## Discussion

**FIGURE 11:**
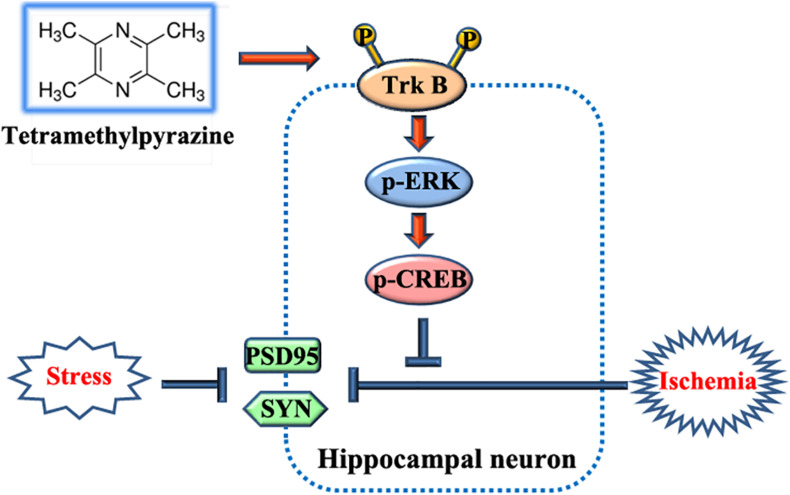
Schematic diagram for the beneficial effects and mechanisms of TMP.

Here, our results delineated that TMP provided multifaceted neuroprotective effects, including relieving depressive and anxious behaviors, improving recognitive ability and sociability, and attenuating learning and memory impairments. The underlying mechanism was that TMP facilitated synaptic rehabilitation such as increasing dendritic length, number, and branches, as well as spine density in rats receiving BCCAO surgery followed by CRS for consecutive 14 days, which we named as the rat BPSD model in the present work (Figure 11). Meanwhile, our *in vivo* and *in vitro* data also illustrated that TrkB/ERK/CREB signaling pathway took part in TMP-induced neuroprotective effect, which was partially abrogated with addition of ANA12 and/or PD98059 (Figure 11).

The rat model established in the present research, which we called the rat BPSD model, is modified based on bilateral common carotid arteries (2-vessel occlusion, 2VO), that mimics the brain disorders of neurodegenerative diseases such as vascular dementia and AD (American Geriatrics Society and American Association for Geriatric Psychiatry, 2003), to physiologically introduce a chronic cerebral hypoperfusion. Here, this model is modified by two steps for ligation of the bilateral common carotid arteries that on one hand decreases the mortality, and on the other hand better simulates the processive ischemic state. Then, Chronic restraint stress (CRS) lasting for 14 days is imposed to rats to introduce psychological impairments. This model, combined with the physiological changes with mental insults, perfectly simulates the pathophysiological process of BPSD, especially for vascular dementia, which is reasonably analogous to the symptoms seen in BPSD (what is referred to as face validity) (McKinney and Bunney, 1969). To our limited knowledge, it is the first report to build a rat BPSD model. Furthermore, two behavioral tests (including OPT and TST) were performed to validate the face validity of this model. Our results showed that CRS alone made rats induce anxiety-like and depression-like behaviors, whilst rats which received BCCAO surgery exhibited slightly more anxious and depressive behaviors, but no significant difference, compared to sham rats. Unlike CRS alone, rats which received BCCAO surgery followed by CRS for 14 days represented severe anxiety-like and depressive behavior. Subsequently, the integrated data from two behavioral tests demonstrated that the rate of susceptibility to BPSD was the highest from rats in the BCCAO/CRS group (75–80%), higher in the CRS group (∼50%), while 20–25% in BCCAO group, and about 15% in the sham group. Herein, the rat BPSD model established in the present study could fulfill the requirements of face validity for an animal model.

Based on the rat BPSD model, we then paid our attention to the hippocampus for the following reasons: (1) chronic cerebral hypoperfusion (CCH) always causes damage to the hippocampus (American Geriatrics Society and American Association for Geriatric Psychiatry, 2003); (2) the majority of cognitive impairments, behaviors, and stress responses are associated with pathology in the hippocampus, in particular, anxiety and depression (Li et al., 2017; Gulyaeva, 2019); (3) abnormity in the hippocampal subfield, especially CA1 and DG, displays a positive correlation between neurodegenerative progression and mental disorders (Haukvik et al., 2018); (4) effective interventions targeting the hippocampus promote reversible behavioral recovery through decreasing neuronal loss or local neuro-vascular network rehabilitation; (5) keeping a sufficient amount and quality of dendric spines is the basis for carrying out functions and avoiding damage in hippocampus (Torres et al., 2018). Our results indicated that BCCAO plus CRS dramatically resulted in a decrease of the number of neurons in the hippocampus owing to a series of causes, like ischemic injury, neuroinflammation, neurotoxicity from glutamate-induced excitotoxicity, etc. Here, our results illustrated that BCCAO plus CRS greatly decreased spine density, dendritic length, dendritic number, and branches in CA1 and DG, which could achieve the requirements for an animal model of construct validity that provides the same anatomic changes in the hippocampus as patients with BPSD (McKinney and Bunney, 1969; Haukvik et al., 2018), while TMP partially reversed this inhibitory effect, thereafter relieving anxious and depressive behaviors, improving recognitive ability and sociability, and ameliorating learning and memory impairments, to some extent. The beneficial outcome triggered by TMP administration is supported by some previous research that TMP and its derivatives provide multiple neuroprotective effects, including preventing neuronal loss *via* attenuating apoptosis (Chen et al., 2017), oxidative stress injury (Zhou et al., 2019), excitotoxicity (Xu et al., 2016), and neuroimmunomodulation through attenuating the release of pro-inflammatory mediators and interferon-γ from microglia and impeding microglial activation in various neurodegenerative disorders including AD and Parkinson’s Disease (PD) (Kim et al., 2014; Pu et al., 2015). Meanwhile, it offers a predictive validity, which is termed as such because treatment modalities effectively reverse the symptoms seen in animals (McKinney and Bunney, 1969) for the rat BPSD model constructed in the present work. Given that the focus of the present investigation is to explore hippocampus-associated mental symptoms, we could not examine the condition of local neurons and microglial activation in hippocampus after BCCAO plus CRS and the effect of TMP on rats received BCCAO plus CRS. Ischemia inevitably induces damage to the cerebral cortex, visual system, whiter matter areas, and permeability of the blood-brain barrier (BBB) (Zhang et al., 2017). Herein, these pathological changes and whether TMP provides neuroprotective effects need to be evaluated in our future research. Additionally, the strategy of establishing a rat BPSD model and the therapeutic effect of TMP on BPSD in the present study could maximally exhibit the pathology and symptoms of BPSD in clinic, which is reflected by face validity, construct validity, and predictive validity. Meanwhile, this strategy is a simple, practical, feasible, and time-saving approach.

To date, numerous therapeutic strategies have been developed to treat BPSD, including non-pharmacological and pharmacological methods. However, the therapeutic effect is still far from ideal due to too many participants related to non-pharmacological interventions, and side effects for drugs administration (Ballard and Corbett, 2010; Kales et al., 2015; Bessey and Walaszek, 2019). Consequently, seeking for suitable candidates, which not only relieve BPSD, but also reduce adverse events, is a feasible way to avail dementia patients against BPSD, and reduces socioeconomic burden to some degree. TMP has been ubiquitously used for the treatment of cerebral infarct due to its neuroprotection through attenuating BBB disruption (Gong et al., 2019), neutrophil activation (Chang et al., 2015), as well as neurogenesis (Xiao et al., 2010), which is consistent with the consequences obtained from our research. Furthermore, previous studies have shown that TMP enhances angiogenesis and blood vessel formation in cerebral ischemia (Gao et al., 2015; Li et al., 2019), which is a supplementary evidence for the neuroprotective role of TMP in pathological anesis in rats suffering from BCCAO/CRS. Amazingly, our results illustrated that long-term usage of TMP did not put weight on rats that received BCCAO/CRS, which overcomes a shortage of weight gain by oral antipsychotic drugs (Atti et al., 2014). Whether administration of TMP could reduce other adverse events, like glucose homeostasis and lipid metabolism, is an interesting issue to answer in the future investigation.

Tetramethylpyrazine holds the capacity of up-regulating the expression level of BDNF to potentiate neurogenesis (Wu et al., 2019) and suppress dendritic spine degeneration (Dong et al., 2018) and local neuroinflammation (Wu et al., 2020), suggesting that its receptor and downstream co-effectors might be involved in TMP improving BPSD in rats with BCCAO/CRS. Our results represented that p-TrkB expression was extensively elevated with TMP administration, which is in line with previous studies (Guo et al., 2018; Kowiañski et al., 2018). Meanwhile, previous studies have attested that activation of TrkB/ERK/CREB signaling pathway elicits neuroprotection through neutralizing oxidative stress and repressing neuroinflammatory response in AD (Zhang et al., 2020), which is coincident with our present results. Moreover, previous studies have proven that TMP could trigger the PI3K/Akt/GSK3b signaling pathway to manipulate neuronal dendritic arborization and spinogenesis in neurodegenerative diseases, including Parkinson’s Disease (PD) and AD (Chen et al., 2017; Hu et al., 2018; Zhou et al., 2019), implying that other signaling pathways, except for TrkB/ERK/CREB, might take part in the neuroprotection induced by TMP. We deciphered the role of the TrkB/ERK/CREB signaling pathway in the current research and provided a therapeutic target for neurodegenerative disorders concomitant with BPSD, particularly for vascular dementia.

Some limitations still exist in the present study. Firstly, approaches need to be developed to overcome the poor solubility and bioavailability of TMP including improvement of dosage form and modification of structural form (Zhao et al., 2016). Next, various administration routes need to be introduced based on improving the bioavailability of TMP.

In sum, the present study establishes a rat BPSD model, combining the physiological changes with mental insults, which maximally simulates the pathophysiological process of BPSD, specifically for vascular dementia. Meanwhile, our data disclose diverse neuroprotective effects of TMP on BPSD including relieving depressive and anxious behaviors, improving recognitive ability and sociability, and attenuating learning and memory impairments, which enlarge the therapeutic scope of TMP in neurodegenerative disorders and provide basic knowledge and feasible candidates for the treatment of BPSD, particularly for vascular dementia.

## Data Availability Statement

The original contributions presented in the study are included in the article/Supplementary Material, further inquiries can be directed to the corresponding author/s.

## Ethics Statement

The animal study was reviewed and approved by Hubei Provincial Hospital of Traditional Chinese Medicine and all procedures were carried out according to the Chinese Animal Welfare Legislation for protection of animals used for scientific purposes.

## Author Contributions

ZT, JQ, and YZ performed most of the experiments, with assistance from QY, XY, JL, and GL. GY and ZT analyzed the results and edited figures. ZT and YZ performed the rat BPSD model and statistical analysis. ZT, XY, and XY performed cell culture and treatments. YZ, ZT, JQ, and HL performed immunoblotting and immunostaining. ZT wrote preliminary draft of the manuscript. HL and GY edited the manuscript and designed experiments and revised the manuscript. All authors approved final version of the manuscript.

## Conflict of Interest

The authors declare that the research was conducted in the absence of any commercial or financial relationships that could be construed as a potential conflict of interest.

## Footnotes

1 http://synapses.clm.utexas.edu
