# Supplementary material for: Tetramethylpyrazine Alleviates Behavioral and Psychological Symptoms of Dementia Through Facilitating Hippocampal Synaptic Plasticity in Rats With Chronic Cerebral Hypoperfusion
Source: Front Neurosci. 2021 May 6;15:646537. doi: 10.3389/fnins.2021.646537 (PMC8134703; doi:10.3389/fnins.2021.646537)
Supplement: Supplementary file 1 [file Data_Sheet_1.DOCX]

**Supplementary Figures and Figure legends**

**Supplementary Figure 1 Tetramethylpyrazine (TMP) exhibits no significant effect on body weight gaining and food intake.**

**(A)** Body weight was measured every three day after BCCAO or BCCAO/CRS in rats. **(B)** The effect of TMP on food consumption after BCCAO or BCCAO/CRS in rats.

**
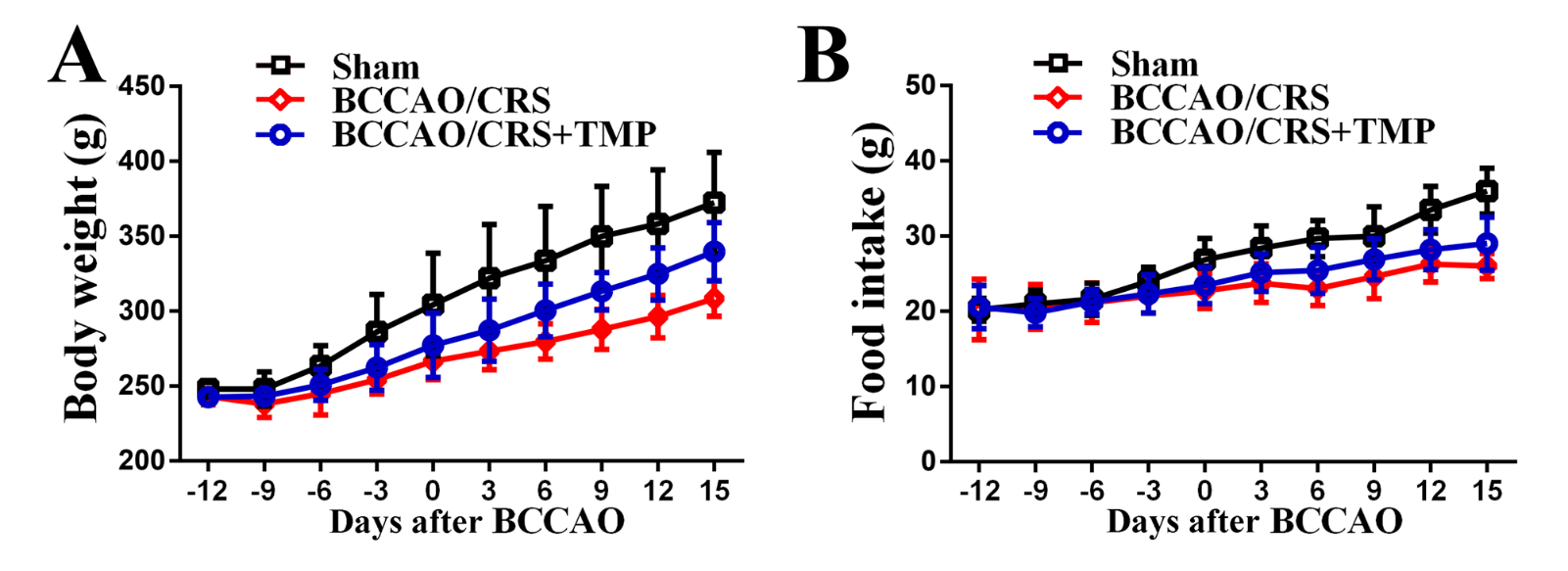
**
